# A CMOS Current-Mode Vertical-Cavity-Semiconductor-Emitting-Laser Diode Driver for Short-Range LiDAR Sensors

**DOI:** 10.3390/s24113513

**Published:** 2024-05-29

**Authors:** Xinyue Zhang, Shinhae Choi, Yeojin Chon, Sung-Min Park

**Affiliations:** 1 Department of Electronic and Electrical Engineering, Ewha Womans University, Seoul 03760, Republic of Korea; zhangxinyue@ewhain.net (X.Z.); rora0414@ewhain.net (S.C.); yeojin1014@ewhain.net (Y.C.); 2 Graduate Program in Smart Factory, Ewha Womans University, Seoul 03760, Republic of Korea

**Keywords:** current mirror, current mode, current steering, LiDAR, VCSEL

## Abstract

This paper presents a current-mode VCSEL driver (CMVD) implemented using 180 nm CMOS technology for application in short-range LiDAR sensors, in which current-steering logic is suggested to deliver modulation currents from 0.1 to 10 mA_pp_ and a bias current of 0.1 mA simultaneously to the VCSEL diode. For the simulations, the VCSEL diode is modeled with a 1.6 V forward-bias voltage and a 50 Ω series resistor. The post-layout simulations of the proposed CMVD clearly demonstrate large output pulses and eye-diagrams. Measurements of the CMVD demonstrate large output pulses, confirming the simulation results. The chip consumes a maximum of 11 mW from a 3.3 V supply, and the core occupies an area of 0.1 mm^2^.

## 1. Introduction

**Figure 1:**
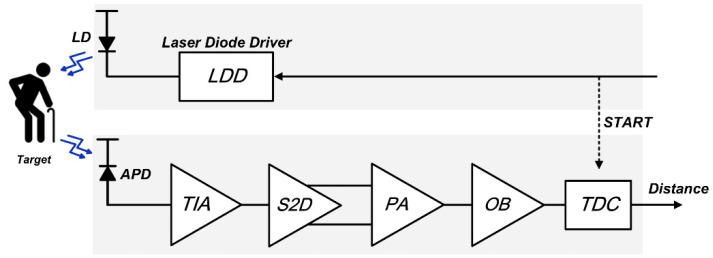
Block diagram of a typical LiDAR sensor.

Recently, light detection and ranging (LiDAR) sensors have been utilized in various fields, such as advanced driver assistance systems for unmanned vehicles, remote sensing detection and navigation systems for robots, and indoor monitoring systems [1,2,3,4,5]. It is well known that most LiDAR sensors exploit the pulsed time-of-flight (ToF) measurement method to ensure successful scanning operations. Figure 1 shows a block diagram of a typical short-range LiDAR sensor, in which the transmitter consists of a laser diode (or VCSEL diode) driver that emits light pulses to targets. Thereafter, the reflected light pulses are detected by the receiver, which comprises an optical detector, a transimpedance amplifier (TIA), a single-to-differential (S2D) converter, a post-amplifier (PA), and a time-to-digital converter (TDC).

Typically, laser diodes are bi-directional and costly. Yet, they require large bias voltages, depending upon the specific target applications (e.g., LiDAR, high-speed optical interconnects, etc.). On the contrary, vertical-cavity-semiconductor-emitting-laser (VCSEL) diodes are unidirectional and operate with much lower bias voltages, which leads to more suitable operations for low-cost, low-power, short-range LiDAR sensors. However, the inevitable bond-wire between the optical device and the front-end integrated circuit causes various design issues, including the notorious problem of voltage headroom, especially in the architecture of DC-coupled laser diode drivers [6,7,8]. Additionally, on-chip electro-static discharge (ESD) protection diodes should be added to avoid damage from the off-chip ESD, which may shrink the receiver bandwidth due to the increased parasitic capacitance.

Previously, a number of studies have been conducted to develop high-speed laser diode drivers implemented in bulk CMOS processes for optical interconnects. Especially for long-range LiDAR sensors in autonomous vehicles, costly laser diode drivers have been incorporated to emit narrow pulses. On the contrary, for short-range LiDAR sensors in indoor monitoring applications, low-cost VCSEL drivers would be more suitable, particularly for the fall detection of dementia patients in their houses.

Conventionally, two types of VCSEL diode drivers exist, i.e., common-cathode and common-anode VCSEL diode drivers. It is well known that a VCSEL diode requires a forward voltage above 1.5 V, and therefore, the supply voltage (V_DD_) must be set high enough to ensure the robust operation of VCSEL diodes. Since the common-anode VCSEL diode can be externally biased with an elevated voltage, the supply voltage of the VCSEL driver chip can be effectively reduced. This configuration substantially reduces power consumption, thus presenting an efficient method to drive VCSELs [9]. Nonetheless, it is crucial to note that the adoption of common-anode VCSEL diodes mandates an additional supply voltage, which is troublesome in practice. Hence, common-cathode VCSEL diode drivers are often preferred due to their compatibility with the prevailing requirements and constraints [10,11,12,13,14].

Section 2 describes conventional voltage-mode VCSEL diode drivers along with the electrical modeling of a common-cathode VCSEL diode, while Section 3 describes the operations of the proposed current-mode VCSEL driver (CMVD). Then, Section 4 presents the simulation and measurement results of the CMVD. Then, our conclusions are presented.

## 2. Conventional Voltage-Mode VCSEL Driver

**Figure 2:**
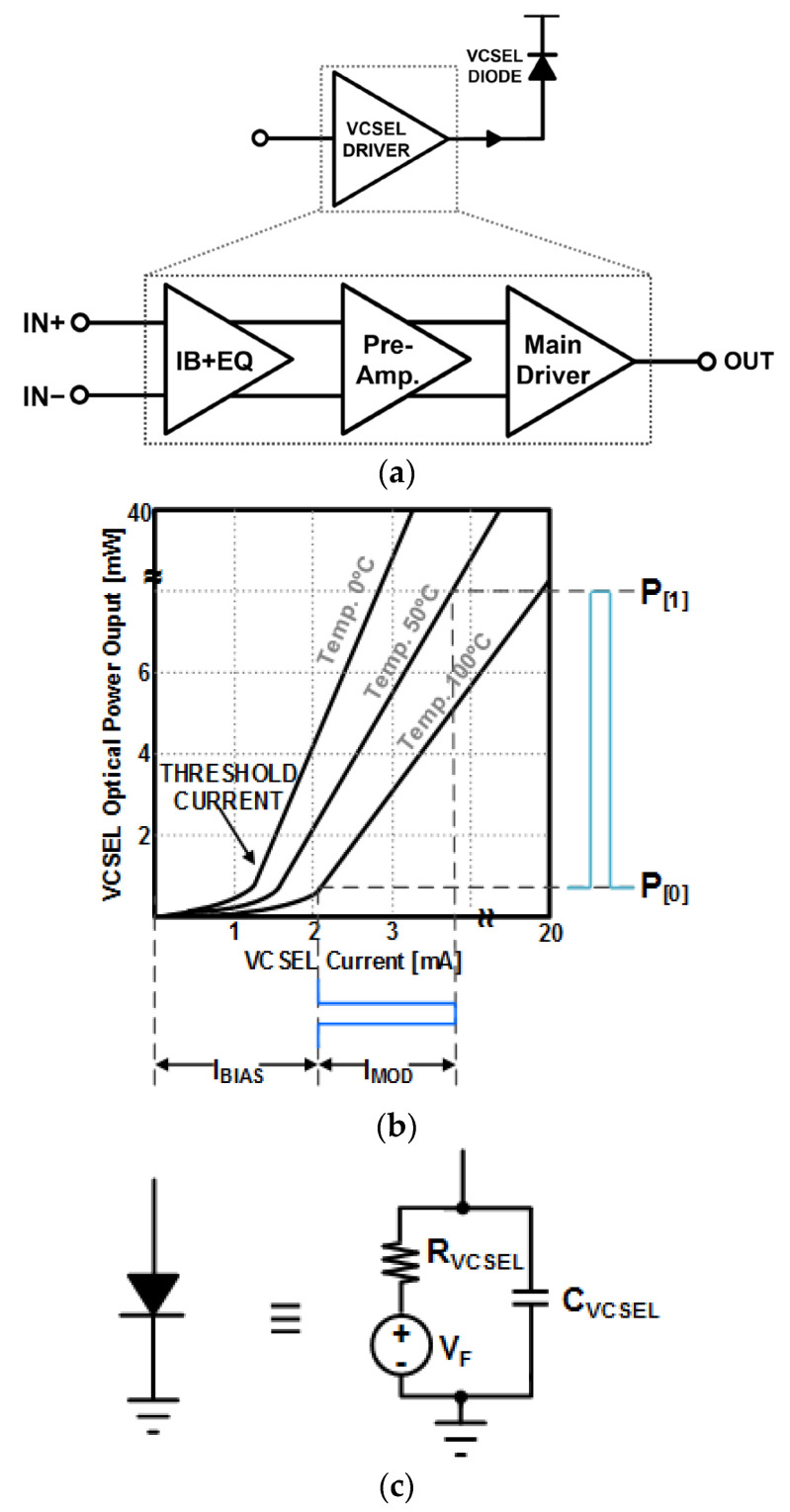
(**a**) Block diagram of a typical VCSEL diode driver, (**b**) VCSEL diode characteristics, and (**c**) equivalent circuit of VCSEL diode for HSPICE modeling [15].

Figure 2a illustrates a block diagram of an optical transmitter where the common-cathode VCSEL diode is driven by a DC bias current (I_BIAS_) and a modulation current (I_MOD_). First, digital logic produces differential digital signals, which then are multiplexed to high-frequency signals by a phase-lock loop (PLL). Here, the front-end VCSEL driver conveys I_MOD_ to a VCSEL diode to transmit optical power. In particular, I_BIAS_ is set to be larger than the threshold current of the utilized VCSEL diode to avoid relaxation oscillation problems while transmitting a logic ‘l’ signal. Also, Figure 2a shows a block diagram of a conventional VCSEL driver that consists of an input buffer for the isolation of the digital logic circuit, an equalizer for bandwidth extension, a pre-amplifier for gain boosting, and a main driver as a current conveyer for the VCSEL diode.

Figure 2b depicts the relationship between the VCSEL diode currents and the corresponding optical power, in which the emitted optical power P[1] is almost linearly increased with respect to IMOD, whereas the bias current (IBIAS) sets the minimum optical power, i.e., P[0]. Namely, the optical power outputs of the VCSEL diode can swing between P[1] and P[0]. The range of the modulation currents should be large enough to generate the desired optical powers even against the low-efficiency VCSEL diode under the high temperature condition of 100 °C. Similarly, the range of the bias currents should be larger than that of the threshold current even under high temperature conditions [15].

Additionally, the VCSEL diode must be modeled as an electrical equivalent circuit so that its transient behavior can be accurately simulated by utilizing HSPICE. Figure 2c shows an example where the VCSEL diode is modeled with a series resistance (R_VCSEL_) of 50 Ω and a forward voltage (V_F_) of 1.6 V, and a parasitic capacitance (C_VCSEL_) of 0.85 pF [15].

**Figure 3:**
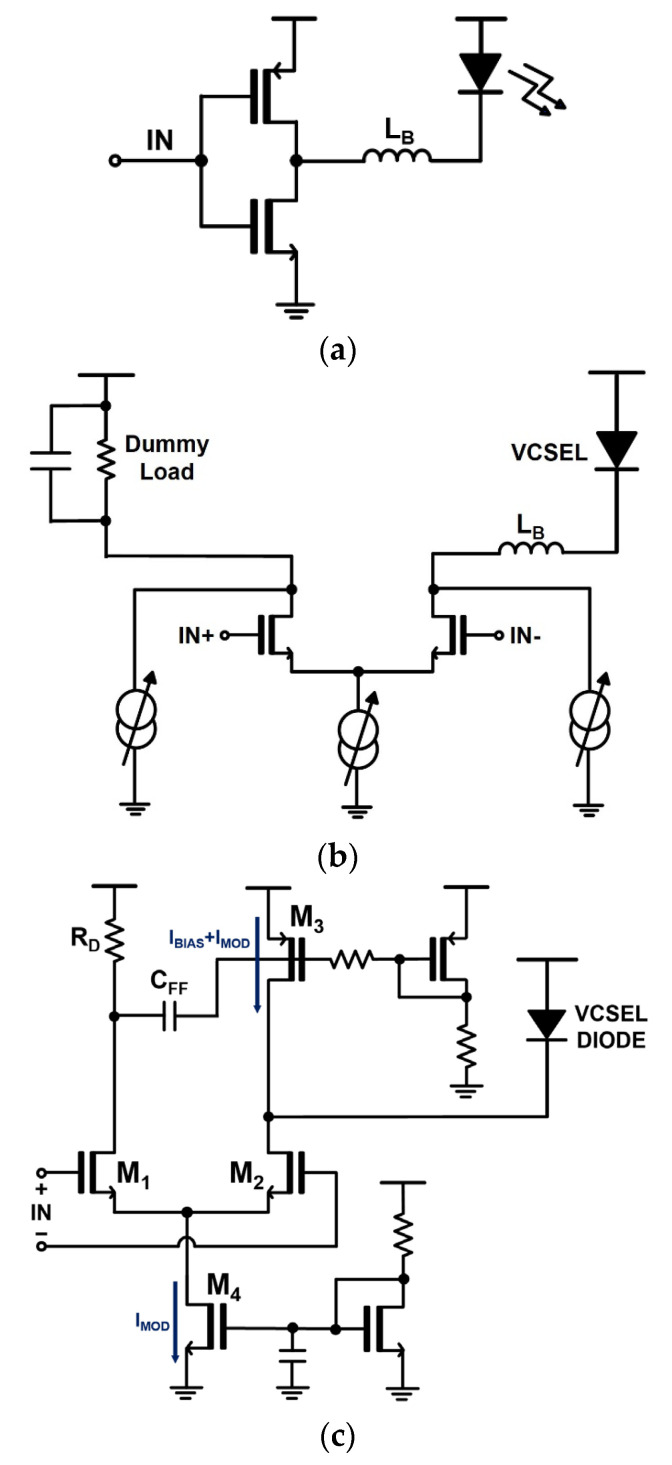
(**a**) Simplified inverter-based laser diode driver, (**b**) differential voltage-mode VCSEL diode driver, and (**c**) voltage-mode VCSEL diode driver with pre-emphasis [15].

Figure 3a illustrates a voltage-mode laser diode driver (VMLD) based on the push–pull inverter scheme, where a current signal is injected into the VCSEL diode through either the pull-up (PMOS) transistor or the pull-down (NMOS) transistor. Although this type of VMLD has been frequently employed for optical transmitters due to its advantageous characteristic of a simple architecture, its differential structure, as shown in Figure 3b, has been preferred for low-common-mode noises due to their symmetry [16]. However, it mandates either an additional laser (or VCSEL) diode connected with a bond-wire for symmetry or an on-chip dummy load to mimic the electrical models of a laser diode including forward-bias voltage, series resistance, and bond-wire inductance. The former raises the manufacturing costs significantly, while the latter deteriorates the symmetry, hence leading to asymmetric output waveforms. Also, the modulation currents of these VMLDs are generated by varying the gate voltages of the current source in the differential pair, which, however, can make it difficult to control the precise amplitudes of the output waveforms. Additionally, the bias control path should be separately equipped to supply the DC bias currents to the laser diode, which requires an extra chip area. If this bias control path malfunctions, it is very likely that relaxation oscillations will occur in the output waveforms.

Figure 3c depicts a schematic diagram of the modified VMLD, which is based on a current-steering circuit. The sum of the bias and modulation currents, i.e., I_BIAS_+I_MOD_, is supplied by a PMOS current source (M_3_), whereas I_MOD_ is controlled by a tail-current source (M_4_). Therefore, with a high input value of ‘1’ at the gate of M_1_ and a low value of ‘0’ at the gate of M_2_, the output current of the VMLD can be the sum of I_BIAS_ and I_MOD_. On the contrary, when M_1_ is turned off with an input value of ‘0’ and M_2_ is turned on, the output current of the VMLD becomes I_BIAS_ only. Moreover, a feedforward technique is employed to improve the rising edges of the output currents by utilizing a high-pass filter with an MIM capacitor (C_FF_ = 3 pF). Consequently, when the input signal rises from ‘0’ to ‘1’, the drain voltage of M_1_ varies abruptly and, therefore, the high-pass filter (C_FF_) allows the high-frequency components to pass through to the gate of M_3_, hence lowering the gate voltage accordingly. This will certainly increase the modulation currents, thereby improving the rising edges effectively. Nonetheless, the aforementioned issues cannot be solved with this voltage-mode configuration.

## 3. Proposed Current-Mode VCSEL Driver

In this paper, a current-mode VCSEL diode driver (CMVD) is suggested to overcome the aforementioned issues. First, the path for I_BIAS_ control is merged with that of I_MOD_ control, thereby reducing the chip area and enabling the stable operation of the VCSEL diode. Second, current-steering logic is exploited to supply various modulation currents to the VCSEL diode, therefore helping to control the amplitudes of the modulation currents better than the VMLDs. Third, the architecture of the proposed CMVD is very simple to design. Yet, there is a disadvantage in this structure in that it has a single-ended configuration. Therefore, it is prone to common-mode noises such as power supply noise. However, this can be reduced considerably by utilizing an off-chip voltage regulator placed on a testing PC-board.

**Figure 4:**
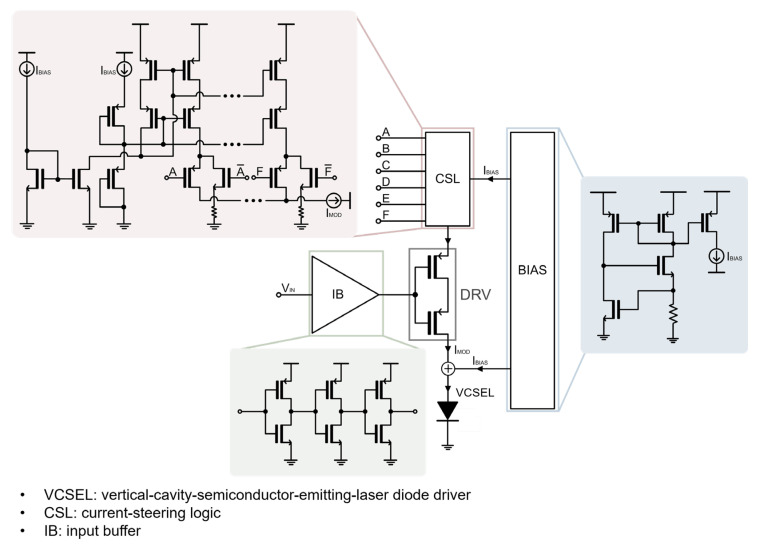
Block diagram of the proposed CMVD.

Figure 4 shows a block diagram of the proposed CMVD, which consists of an input buffer (IB) for isolation from the preceding stage, a driver circuit (DRV) for passing through the bias current and the modulation currents to the VCSEL diode, a bias circuit for the generation of the bias currents, current-steering logic (CSL) for the supply of the varying modulation currents via a 6-bit (A~F) control digital-to-analog converter (DAC), and a VCSEL diode.

**Figure 5:**
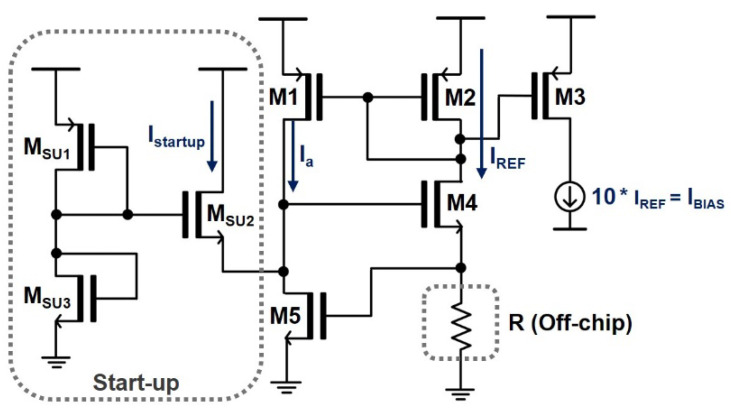
Schematic diagram of the proposed BIAS circuit.

Figure 5 depicts a schematic diagram of the BIAS current control circuit, where the currents (I_a_ and I_REF_) flowing through the two branches are identical due to the action of the current mirrors formed by the PMOSs (M_1_ and M_2_). Then, I_REF_ flows through a resistor R, which produces a voltage drop, i.e., I_REF_ x R, that is also the gate-source voltage V_GS5_ of M_5_. The NMOSs (M_4_ and M_5_) function as regulated cascodes to stabilize the bias current via the feedback mechanism. At this point, I_REF_ barely varies with the supply voltage, providing good stability. Once a stable reference current is achieved, another PMOS (M_3_) mirrors the DC current of 1 mA to the following CSL circuit and the VCSEL diode.

The PVT (process, voltage, temperature) variation simulations of the proposed BIAS circuit confirm that the modulation currents alter 21% at maximum for the worst case of SS with a supply voltage of 2.97 V at a temperature of 125 °C. This reveals that the BIAS circuit provides quite stable operations against the considerable PVT variations.

**Figure 6:**
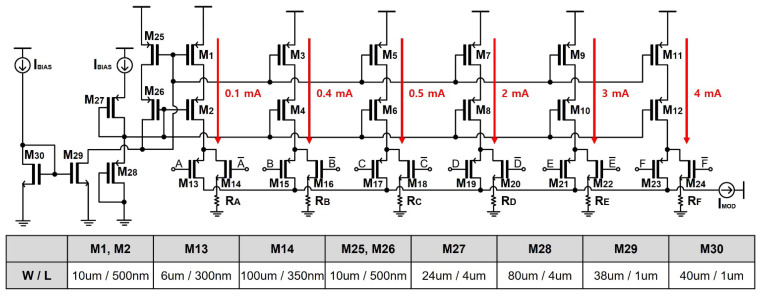
Schematic diagram of CSL circuit.

Figure 6 shows a schematic diagram of the CSL circuit, where the bias current is inserted to the NMOS current mirror (M_8_, M_9_). Then, it is mirrored by utilizing parallel PMOS current mirrors (M_1_~M_7_, M_1C_~M_7C_) that can be turned on and off with respect to the input DC voltages of A through F. The diode-connected PMOS transistors (M_10_, M_11_) set the DC voltages of the PMOS current mirrors. It is noted that the DC currents of the PMOS current mirrors pass through to the VCSEL diode when the gate voltage is lower than the threshold, i.e., V_DD_ – |V_THP_|. Here, V_DD_ represents the supply voltage and V_THP_ is the threshold voltage of a PMOS transistor.

The PVT variation simulations of the proposed CSL circuit confirm that the modulation currents alter 20% at maximum for the worst case of SS with a supply voltage of 2.97 V at a temperature of 125 °C. This reveals that the CSL block also provides stable operations against the considerable PVT variations.

## 4. Layout and Simulation Results

**Figure 7:**
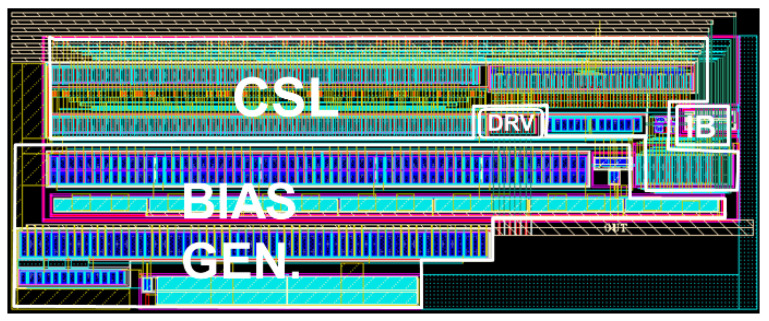
Chip layout of the proposed CMVD.

Figure 7 illustrates the layout of the proposed CMVD, where the chip core occupies an area of 0.1 mm^2^. Post-layout simulations were conducted for the proposed CMVD by using the model parameters of standard 180 nm CMOS technology. The DC simulations show a maximum power dissipation of 36.3 mW from a 3.3 V supply.

**Figure 8:**
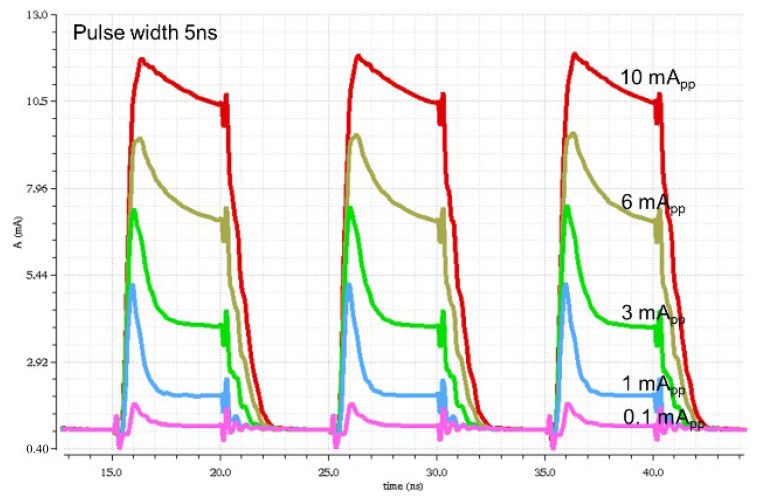
Simulated pulse responses of the proposed CMVD with modulation currents of 0.1 ~ 10 mA_pp_ for a narrow pulse width of 5 ns.

Figure 8 depicts the simulated pulse responses of the proposed CMVD for a narrow pulse width of 5 ns with variations in the modulation currents from 1 mA_pp_ to 10 mA_pp_. It is clearly seen that the output pulses are linearly transmitted with the variation in the modulation currents. The peaking at the rising edges might be attributed to the parasitic capacitance at the output node, which includes the capacitance of the VCSEL diode and that of the CSL circuit.

**Figure 9:**
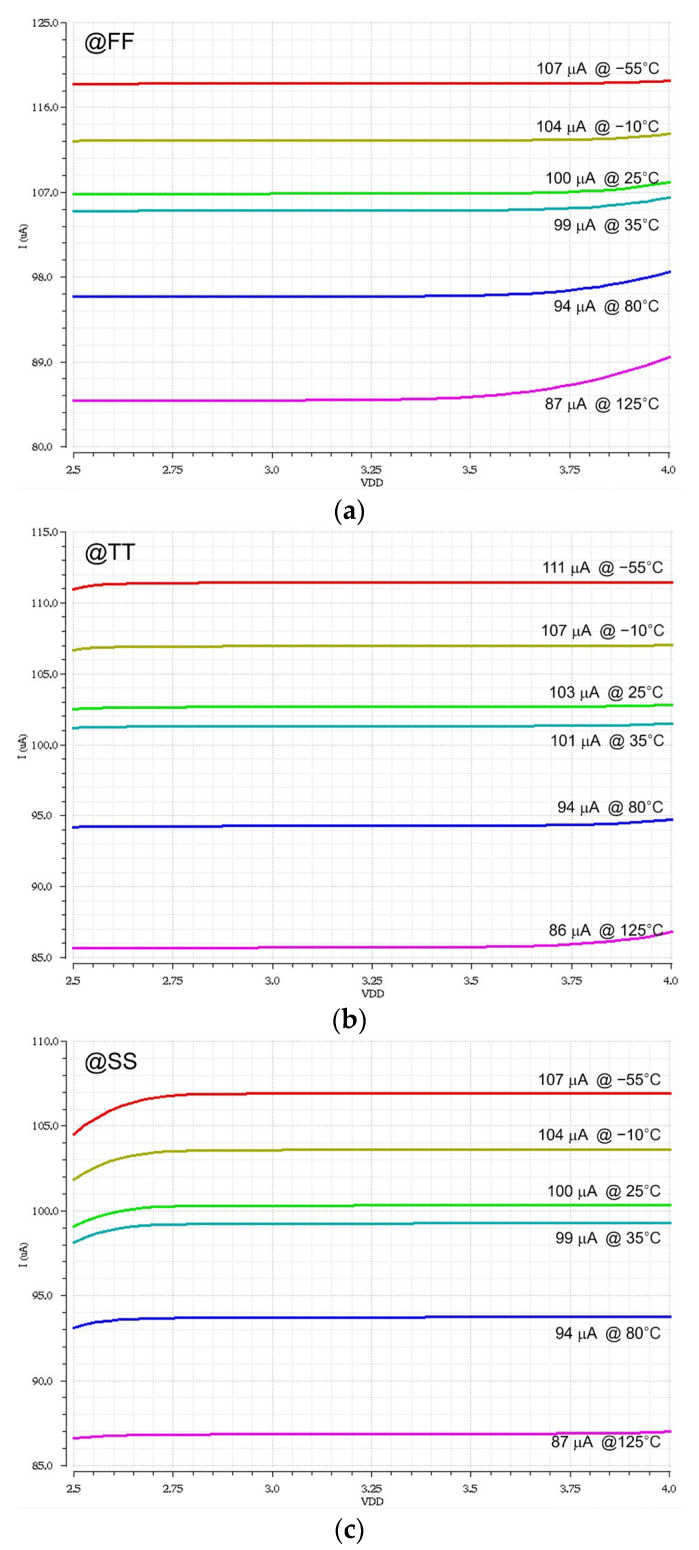
PVT variation simulations of the proposed CMVD, where the *x*-axis is V_DD_ and the *y*-axis is the bias currents for the (**a**) FF corners, (**b**) TT case, and (**c**) SS corners.

Figure 9 shows the PVT variation simulations of the proposed CMVD for the bias current with three different corners in more detail, in which the *x*-axis represents the supply voltage, which varied from 2.5 V to 4.0 V, whereas the *y*-axis shows the bias currents at various temperatures ranging from −55 °C to 125 °C. In the case of the FF corner shown in Figure 9a, the largest deviation of 13% occurs at 125 °C when compared to the reference current of 100 µA at 25 °C. In the case of the SS corner in Figure 9c, the same deviation of 13% occurs at 125 °C.

**Figure 10:**
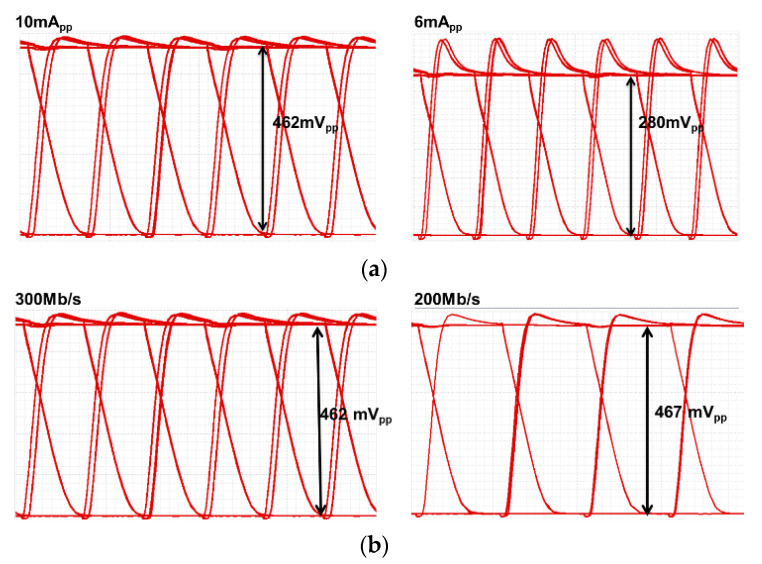
Simulated eye-diagrams of the proposed CMVD for (**a**) different modulation currents of 10 mA_pp_ and 6 mA_pp_ at 300 Mb/s, and (**b**) 300 Mb/s and 200 Mb/s at a 10 mA_pp_ current.

Figure 10a shows the simulated eye-diagrams of the CMVD at the same 300 Mb/s speed with different input current levels of 10 mA_pp_ and 6 mA_pp_. These results confirm that the output amplitudes are linearly amplified with respect to the modulation currents. Figure 10b depicts the simulated eye-diagrams at the same input current levels of 10 mA_pp_ at different speeds of 300 Mb/s and 200 Mb/s.

## 5. Measured Results of the Proposed CMVD

### 5.1. Experimental Methods

**Figure 11:**
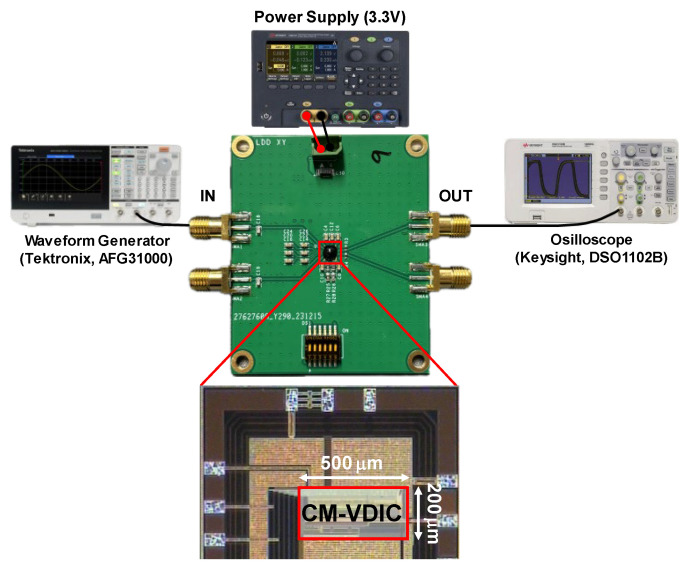
Chip photo and test setup for the proposed CMVD.

Test chips of the proposed CMVD were implemented in a standard 180 nm CMOS process. Figure 11 shows a chip microphotograph and the PC-board module used for testing, where a waveform generator (Tektronix AFG31000) was utilized to drive the CMVD, and a VCSEL diode operated at an 850 nm wavelength for optical measurements. The output pulses were measured by an oscilloscope (Keysight DSO1102B).

### 5.2. Measured Results

**Figure 12:**
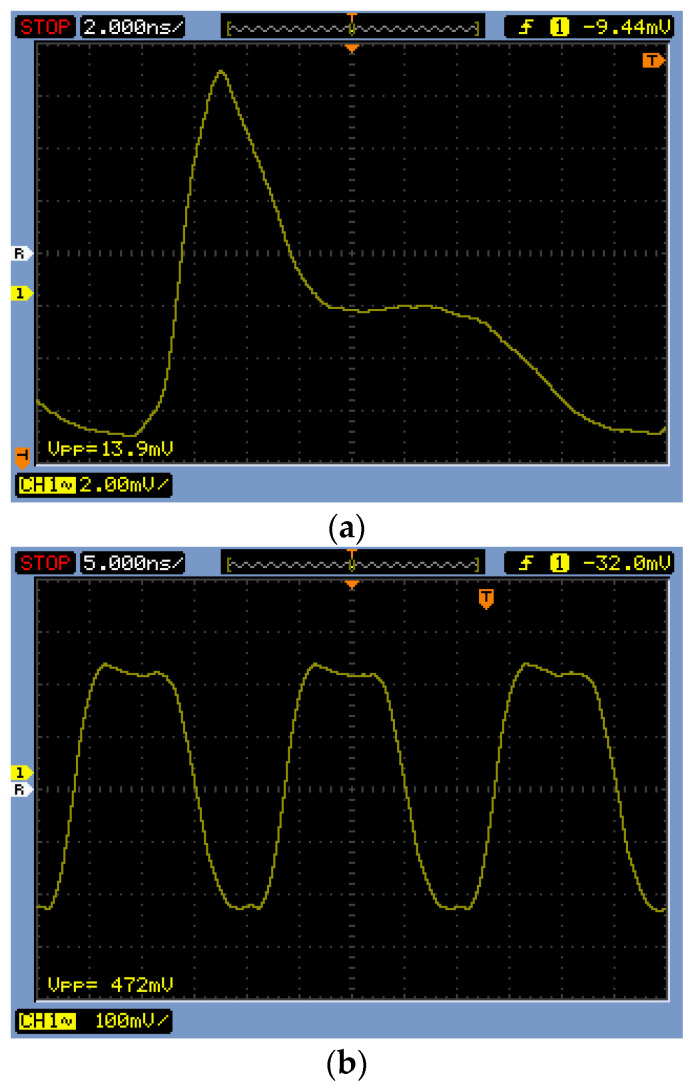
Measured results of the CMVD (**a**) with 0.1 mA_pp_ output current and (**b**) with 10 mA_pp_ output current.

Figure 12 demonstrates the measured results of the proposed CMVD chip at a pulse width of 10 ns with 0.1 mA_pp_ and 10 mA_pp_ output currents. It clearly shows that the CMVD provides modulation currents of 0.1~10 mA_pp_.

Table 1 summarizes and compares the performance of the proposed CMVD with the previously reported CMOS laser (or VCSEL) diode drivers. Ref. [9] suggested a 12-channel laser diode driver array, where each channel could provide modulation currents of 1~16 mA_pp_ with variable bias currents of 1~10 mA. Still, high power consumption was indispensable. Ref. [12] presented a voltage-mode VCSEL driver where optical eye-diagrams of all four channels were measured at 10 Gb/s. Yet, the chips were produced in a costly 65 nm CMOS. Ref. [13] presented a differential push–pull voltage-mode VCSEL driver implemented in a 65 nm CMOS process with a limited modulation current of 7 mA. Ref. [14], on the other hand, demonstrated a CMOS linear VCSEL driver for an intermediate frequency over fiber links, where a large bias current of 10 mA was required. In this work, we proposed a novel single-ended CMVD with stable bias currents by exploiting regulated cascode current-mirror circuitry and with a novel CSL circuit to provide variable modulation currents up to 10 mA_pp_.

## 6. Conclusions

We have presented a current-mode VCSEL driver in this paper, in which a current-steering logic circuit utilizing a wide-swing cascode current-mirror topology was employed to facilely deliver modulation currents with a PMOS transistor array. By turning on/off the gates of A to F, the CSL circuit can transmit a minimum modulation current of 0.1 mA_pp_ and a maximum modulation current of 10 mA_pp_, respectively. Also, a bias current generator using a regulated cascode circuit was exploited to provide stable DC bias currents (i.e., 1 mA in this work) against the notorious PVT variations. The post-layout simulations not only demonstrated large output pulses, but also revealed small PVT variations (13%) against a wide range of temperatures from −55 °C to 125 °C, even at the worst corners. The measured results of the proposed CMVD confirmed the simulation results. The whole CMVD chip consumed a maximum of 11 mW from a single 3.3 V supply, and the chip core occupied a small area of 0.1 mm^2^. To conclude, the proposed CMVD produced via a low-cost 180 nm CMOS process could be a highly efficient solution for application in low-power, low-cost, short-range LiDAR sensors.

## Figures and Tables

**Table 1:** Performance comparison with previously reported CMOS laser (or VCSEL) diode drivers.

Parametars	[9]	[12]	[13]	[14]	This Work
CMOS technology (nm)	180	65	65	65	180
Supply (V)	1.8	1.2/2.5	1.05	1.2	3.3
Laser diode	laser diode	VCSEL	VCSEL	VCSEL	VCSEL
Configuration	voltage mode	voltage mode	voltage mode	voltage mode	current mode
Output signaling	differential	single-ended	differential	differential	single-ended
Driver type	common anode	common cathode	common cathode	common cathode	common cathode
Modulation current (mA_pp_)	1~16	6	7	2.5	0.1~10
Bias current (mA)	1~10	2	N/A	10	1
Max. power dissipation (mW)	98	34	24.2	15	11
Core area (mm^2^)	0.13 *	3.2 (chip)	0.1 *	4 (chip)	0.1 *

* single-channel.

## Data Availability

Data are contained within the article.
